# Myocardial Infarction and Stroke Risk in Young Healthy Men Treated with Injectable Testosterone

**DOI:** 10.1155/2015/970750

**Published:** 2015-06-01

**Authors:** Robert S. Tan, Kelly R. Cook, William G. Reilly

**Affiliations:** ^1^Low T Institute, Dallas, TX 76092, USA; ^2^University of Texas, Houston, TX 77030, USA; ^3^Baylor College of Medicine, Houston, TX 77030, USA; ^4^Michael DeBakey VAMC, Houston, TX 77030, USA; ^5^Opal Medical Clinic, Houston, TX 77098, USA

## Abstract

This study was conducted to examine the association between testosterone therapy and new myocardial infarction (MI) and stroke events in a series of patients treated at Low T Centers across the United States, consisting of mainly young (mean age = 46), otherwise, healthy men. Electronic medical records were queried between the years 2009 and 2014 to identify patients diagnosed with hypogonadism, MI, and stroke, as indicated by ICD-9 codes. The incidence of MI and stroke events was compared to community-based registries. 39,936 patients recruited from 40 Low T Centers across the United States were treated and 19,968 met eligibility criteria for receiving testosterone treatment. The incidence rate ratio (IRR) for MI in testosterone- (T-) treated versus nontreated patients was 0.14 (C.I. = 0.08 to 0.18, *P* < 0.0001) whereas the IRR for stroke for T-treated versus nontreated patients was 0.11 (C.I. = 0.02 to 0.13, *P* < 0.0001). There was no evidence of worsening preexisting MI or stroke in patients treated with testosterone. The experience in Low T Centers shows that, in an injectable testosterone patient registry, testosterone is generally safe for younger men who do not have significant risk factors. Of patients that developed MI with testosterone, there was no association with testosterone or hematocrit levels.

## 1. Introduction

In the past year, concerns have been raised over the safety of testosterone replacement therapy (TRT) because of two peer-review papers associating myocardial infarctions (MI) and strokes with TRT use by men [1, 2]. These studies have been followed by a flurry of potential litigation against manufacturers of testosterone. In addition, great confusion has arisen for both patients and their treating physicians.

In the first article by Vigen et al. [2], the authors initially excluded 1132 men from analysis who had received a testosterone prescription after experiencing an event (MI or stroke) but, later, published an erratum in 2014 disclosing that the number of patients excluded should have been 128, not 1132, resulting in an 89% error rate. Among the original group of 1132 excluded individuals, 100 patients were in fact women, not men. Moreover, an additional exclusion criterion based on either missing coronary anatomy data or data classification as “other” was incorrect and changed from 1301 to 397 patients. Despite strong media attention on these findings, these clear inconsistencies in data reporting undermine the credibility of these findings.

In the 2014, article by Finkle et al. [1], the authors compared nonfatal MI among men prescribed testosterone versus PDE5 inhibitors (PDE5Is) for treatment of hypogonadism. A critical limitation of this study was that testosterone levels of men prescribed PDEs were unknown, either at baseline or after treatment. The study relied on insurance data; patients were on variable treatment protocols not defined in the study. The authors compared a group of men with presumably low testosterone who may not have received adequate treatment for hypogonadism against an unrelated cohort of men with unknown but presumed average testosterone levels. Therefore, two treatment groups were not comparable and the interpretation of the study was limited.

The release of these two peer-review articles prompted the US Low T Center to initiate an internal quality management program to determine whether its patients were adversely impacted by higher risk of MI or stroke following initiation of testosterone treatment. The Low T Centers are a privately owned group of 50 clinics distributed across the United States [3]. These clinics are staffed by board certified physicians in various specialties as well as physician assistants. The protocols for determining treatment are specific to Low T Centers, modified from established guidelines from both the Endocrine Society and the American Association of Clinical Endocrinologists [4, 5]. Patients are selected for treatment if deemed hypogonadal, defined as total testosterone <350 ng/dL or free testosterone <10 ng/mL. In addition, patients cannot have contraindications such as prostate cancer, breast cancer, polycythemia, severe obstructive sleep apnea, and/or severe untreated lower urinary tract symptoms (LUTS). Approximately, 19,968 of 40,000 (50%) patients that seek treatment in these Low T Centers do not qualify after screening tests. Among those who do qualify, they must undergo supervised, short acting injection treatments, requiring clinic visits every one to two weeks. During these visits, additional clinical parameters such as blood pressure, testosterone, and estradiol levels are closely monitored.

This study consists of an examination of the incidence of myocardial infarction (MI) and/or stroke in a large, multicenter practice (Low T Centers) with uniformity in treatment protocols and adherence to standards of care due to concerns for the safety of patients taking testosterone within such large, multisite practices.

## 2. Subjects and Methods

**Figure 1:**
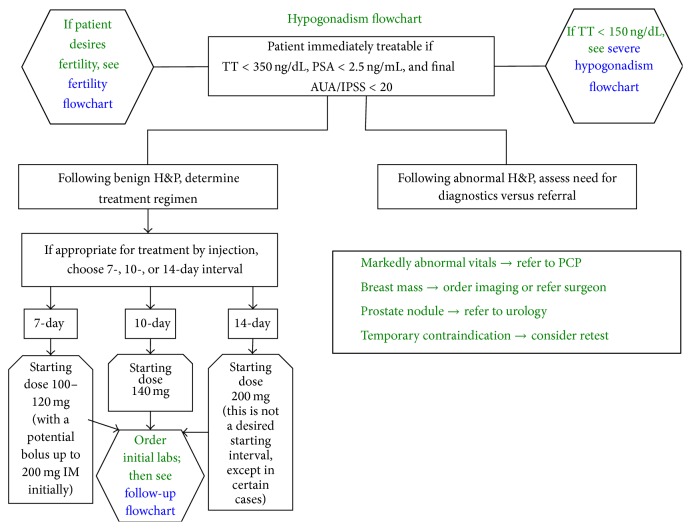
Flow diagram for patient enrolment protocol in a Low T Center.

**Figure 2:**
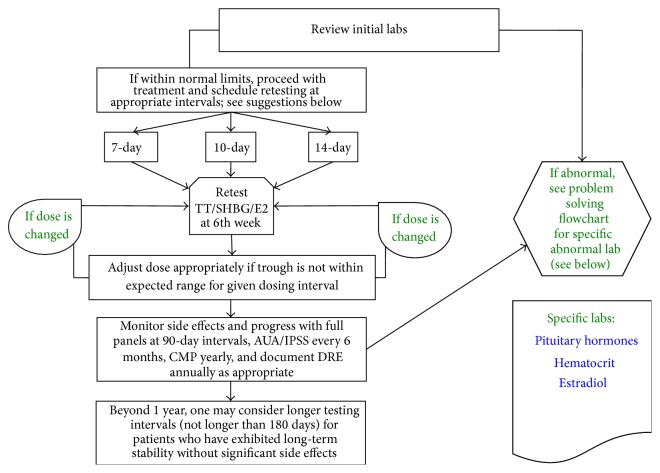
Flow diagram for follow up patients at a Low T Center.

This study consisted of a retrospective, multicenter medical chart review across 40 participating US Low T Centers, with a geographical concentration in Texas. At these centers, patients received mainly intramuscular testosterone cypionate once weekly or every 2 weeks. Blood draw was every 90 days but may be earlier based on clinical indications (Figures 1 and 2). Prior to systematic data extraction, three investigator conference calls were held with Low T Center providers to ensure that all International Center for Diseases- (ICD-) 9 codes were updated, with particular attention to MI and stroke. Investigators also interviewed families with patients that had sudden deaths, presumed to be fatal MI. The principal investigator at each site also underwent training with the information technology specialist at his/her respective Low T Center, including training on accessioning the patient's electronic health record (EHR). Researchers also underwent Good Clinical Practice (GCP) training with the Food and Drug Administration. Approval was obtained from the Aspire Institutional Review Board (IRB) to conduct the study using EHR chart reviews. Each research site captured deidentified data on case report forms using numeric codes to ensure patient confidentiality.

The medical records of all male patients evaluated in the Low T Centers between October 1, 2009, and March 15, 2014, were queried for a diagnosis of hypogonadism, myocardial infarction, and stroke using the following ICD-9 codes: 257.2, 412, 410.80, 438.89, and 436. In order to be diagnosed with hypogonadism, the patient was required to have reported symptoms in addition to having had a serum testosterone level below 350 ng/dL. This cut-off was determined by the protocol committee at Low T Centers. Records for patients initiating testosterone replacement therapy (TRT) and having blood testing for hormone assays were reviewed. Inclusion in the study required availability of full demographic information, male gender, age ≥ 20 years, and a history of at least one visit to a Low T Center, whether treatment was received or not. All data were recorded in an Excel spreadsheet with patient information linked to a unique identifier to maintain patient confidentiality. For baseline and follow-up visits, the visit date, ICD-9 codes, and adverse events for those on TRT were recorded from the ICD-9 diagnoses available in the EHR system “Advance MD” [6]. Of the patients having a record of MI or stroke, the practitioners were advised to contact the patient for further interview. Total testosterone levels were drawn in the clinic and results were given to the patient within 30 minutes. Testosterone assays were performed primarily by the Qualigen FastPack IP TestoImmunoassay, which is a chemiluminescent immunoassay for the in vitro quantitative determination of total testosterone in human serum. Estradiol levels were measured using electrochemiluminescence immunoassay (ECLIA) through Lab Corp. Free testosterone levels were calculated from total testosterone and sex hormone binding globulin (SHBG).

The statistical significance of between-cohort differences in categorical variables was tested using the chi-square test and for continuous variables using the two-sample Student's *t*-test. All tests were two-tailed with a significance level of *P* < 0.05. Analyses were conducted using SPSS and SYSTAT.

This study focused on five comparison registries for MI incidence [7–10]. As patients in Low T Centers represented primarily commercial health insurance patients, the Kaiser Permanente database in Northern California (which was also mainly commercial insurance) was chosen for comparison. Since Low T Centers are distributed across the US, an additional 4 registries were included for comparison from different parts of the country. No single registry with a predominantly younger population could be identified. The average rate of MI from the other four combined US-based registries was 203 events per 100,000 persons which approximates that of the Kaiser site at 208 events per 100,000 persons. Rates of MI varied from 71.6 to 241 events per 100,000 persons. The Northern Manhattan Registry data were selected for comparison of stroke events because of the known rigor in data collection and quality [11].

## 3. Results

### 3.1. Patient Population

Data were extracted from the electronic health records (EHR) of the 40 medical centers across the United States. All subjects were male and the age range was 20 to 86 years, with 87% of patients being less than 55 years old and only 3% being greater than 65 years old. From a total population of roughly 40,000 patients, this study contributed to up to 160,000 person-years of observation. The characteristics of the study population, as well as the comparable registries, are summarized in Table 2. Anthropometric measurements were not completed in some Low T Centers and, as such, not reported in this publication.

### 3.2. Treatment Outcomes

**Figure 3:**
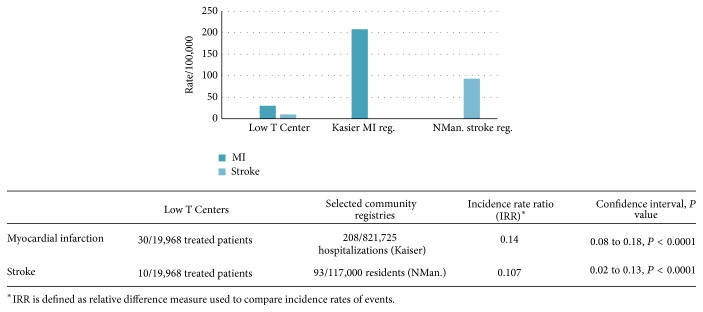
Summary of cardiac events in Low Testosterone (T) Centers and comparison registries (per 100,000 persons).

The rate of MI and stroke varied across the country. A summary of these rates is presented in Table 1. Between years 2009 and 2014, 39,937 patients were seen and approximately 50% met criteria for treatment. Of the treated patients, there were 4 nonfatal MI and 2 probable fatal MI; thus the rate of new MI was 30 events per 100,000 persons. The 2 probable cases were presumed to be from MI because of sudden death; since no postmortems were performed and the 2 deaths could have been from other causes, they were still included in the analysis. There were 46 patients with pretherapy MI of which none had adverse outcomes after testosterone. Of the treated patients, there were two cases of stroke; thus, the rate of new strokes was 10 events per 100,000 persons. There were 12 patients with pretherapy stroke and none had adverse outcomes after testosterone. The risks for new MI and stroke were compared to the Kaiser Permanente and Northern Manhattan Registry which were 208 events per 100,000 persons and 93 events per 100,000 persons, respectively. As the treated population base at Low T Centers was 19,968 patients and, to match the controls, the rate was given a multiple of 5 times, thus, the incidence rate ratio (IRR) for MI in testosterone-treated patients is 0.14 (C.I.: 0.08 to 0.18, *P* < 0.0001) whereas strokes is 0.11 (C.I.: 0.02 to 0.13, *P* < 0.0001) (Figure 3).

Overall, the mean time of exposure to testosterone for all patients in our study was 17 months. Among those with cardiovascular events, there appears to be no association of time of MI or stroke with the duration of exposure to testosterone. MI and stroke occurred at various times of exposure, ranging from 4 weeks to 4 years. In a particular case of a 55-year-old patient, the cardiac event occurred after 6 weeks of treatment, with the observation that both testosterone and estradiol rose 3 times from baseline. The association of high estradiol from aromatization from testosterone to the cardiac event is likely serendipitous; but there will be further analysis of this data, which will be beyond the boundaries of this publication. The results are summarized in Table 3.

The mean total testosterone levels of all patients were determined. For patients aged 25 and younger, the level was 432 ng/dL; for patients aged 25–44 years, the level was 460 ng/dL; for patients aged 45–65, the level was 503 ng/dL; and, for patients above 65 years, the level was 552 ng/dL (data not shown). Measurements of the mean total testosterone level could occur at the beginning, middle, or end of the injection cycle. As such, the range of total testosterone varied from 50 to 1600 ng/dL. However, the mean testosterone level of all treated patients irrespective of age was 543 ng/dL.

The Low T Centers see patients that are generally much younger than in a typical urological or endocrinology practice. The mean initial total testosterone level of those that received testosterone treatment and who were below 25 years was 178 ng/dL. The typical patient is often self-referred rather than being referred from another physician. Also, the approach may be more preventive. Of the patients seen at the Low T Centers, the prevalence rates of hypertension, diabetes, and hyperlipidemia were 15%, 4%, and 12%, respectively. Comorbidities were reported by the patient and confirmed by the treating provider and entered in the problem list. The population seen at the Low T Centers contrast from a typical academic andrology practice, where there may be a higher prevalence of comorbidities [12].

The levels of testosterone were studied in patients with MI. Of the patients that had MI, the last mean total testosterone was 480.50 ng/dL and was lower than the mean total testosterone of all treated patients, which was 543.0 ng/dL.

## 4. Discussion

In the past, most of the literature supported the role of endogenous testosterone in protecting the cardiovascular system [13, 14]. In some small interventional studies, testosterone has been shown to have vasodilatory properties which implied cardioprotection [15]. In epidemiological studies, elevated blood pressure is an established risk factor for heart attacks and strokes. Blood pressure was found to be inversely correlated to testosterone levels [16]. Studies also show that testosterone suppression leads to accelerated atherosclerosis [17].

However, two recent papers found an increased risk of MI and strokes [1, 2]. This caused widespread concerns among practitioners who prescribe testosterone use. In fact, practitioners were so concerned that a group of experts formed the Androgen Study Group to address the findings of these studies [18]. Moreover, several important challenges to these findings have since been published [19–21].

In this study, it was observed that the rates of MI and stroke in a multicenter practice were very low, in contrast with these two recent papers [1, 2]. Instead of associating TRT with MI and stroke, the Low T Center data paradoxically demonstrated that TRT may be associated with lower risk of MI events, when compared to 5 national registries (Table 1). The observed MI incidence rate of 30 events per 100,000 persons was lower than any rate reported in the United States; the lowest rate was seen in the New York State Registry, which included 71.6 events per 100,000 persons. The Fukushima registry had an event rate of 37.9 per 100,000, despite stressors from the nuclear leak, though Japanese men are known to have lower rates of MI [10].

Unfortunately, the patients that did not qualify for testosterone therapy in the Low T Centers were not followed up and, therefore, could not be examined as controls. This study also relied on patient and family reporting of MI/stroke; this could have resulted in under- or overreporting, thereby possibly limiting the interpretation of these findings. Adverse outcomes were verified by interview and retrieval of medical records. The registries used as comparison cohorts do have limitations as they are not ideally matched controls. Admittedly, the Low T Center population is younger and has a lower prevalence of risk factors associated with cardiovascular events, such as hypertension, diabetes, and hyperlipidemia. To our knowledge, there are no similar low-risk and age-matched patient registries. It may be possible that patients seeking testosterone therapy are healthier, exercise more, and eat better than the general population. However, what is undeniable is that, even for a large practice of this size, treating only hypogonadism, very few cases of MI and stroke were reported.

Both Vigen et al. and this study performed retrospective chart review. However, only Veterans in the VA system were included in Vigen et al.'s article. These were not just Veterans but very ill patients with suspicion of heart disease, requiring the need for cardiac catheterization to elicit whether they had blocked coronary arteries. In the Low T Centers, most patients were healthy with few comorbid conditions, as implied by the low prevalence of risk factors for heart disease for hypertension (15%), diabetes (4%), and hyperlipidemia (12%) relative to the Veteran population [22]. In addition, the current study population was approximately 15 times the size of the Vigen et al. study, lending greater power to examine the study questions. There is a well-known limitation of studies using ICD-9 codes. The paper by Vigen et al. used ICD-9, but their data may not have been accurately classified. Oddly, some women were included in this study. In this present study, to avoid such problems, three nationwide telephone conferences groups were held with all providers, educating them on the correct codes required, to review all patients and update the ICD-9 as appropriate. Curiously, the mean level of total testosterone in Vigen et al.'s study was 332 ng/dL, and, by some definitions, this level reflects undertreatment or even a hypogonadal state. In contrast, the present study observed a mean of 543 ng/dL for treated patients. National guidelines for treatment of hypogonadism were followed. It could be argued that the patients with lower mean total testosterone levels achieved in the Vigen et al. study were themselves a risk for MI and stroke. Mean age of patients in the Vigen et al. paper was 63 years. In contrast, only 3 percent of the Low T Center patients were above 65 years.

Limitations to the Finkle et al. study include short duration, limited to 90 days, making it difficult to draw firm conclusions over time. In the Low T Centers, the follow up was up to 5 years. The Finkle et al. study was based on insurance data and included a heterogeneous group of patients using different treatment protocols. In contrast, treatment protocols at the Low T Centers are standardized throughout the different US centers. Testosterone levels were not reported in this study, which made us query whether these patients were actually treated or even reached eugonadal levels. The Low T Centers treat only men removing potential gender misclassification.

Baillargeon et al. [23] studied testosterone therapy in older men and examined the risk of MI in a population-based cohort of older men receiving intramuscular testosterone. They used a 5% national sample of Medicare beneficiaries. 6355 patients treated with at least 1 injection of testosterone were matched to a cohort of testosterone nonusers at a 1 : 3 ratio. They found that receiving testosterone therapy was not associated with an increased risk of MI (hazard ratio [HR] = 0.84; 95% C.I. = 0.69–1.02). But, for men in the highest quartile of the MI prognostic score, testosterone therapy was associated with a reduced risk of MI (HR = 0.69; 95% C.I. = 0.53–0.92). The similarity of these findings with the present study was that injectable testosterone was used. However, this study had younger patients with fewer comorbidities. In addition, this study followed a similar protocol of treatment. It may be argued that testosterone can protect from MI and strokes, if these criteria of injectable therapy and a fixed protocol were followed for a younger population.

Corona et al. [24] performed a meta-analysis of placebo controlled randomized controlled trials on the effect of testosterone on sexual function. No difference in the incidence of cardiovascular diseases was reported. When testosterone was compared to placebo for a comparison of cardiovascular diseases, the HR was 0.606 (95% C.I. = 0.157–2.335).

A review by Carson and Rosano [25] looked at available clinical trial data, indicating that the use of testosterone in middle-aged to elderly men does not increase cardiovascular risk nor does it unfavorably modify cardiovascular risk profile. They concluded that prospective data from large, well-designed, long-term trials of testosterone treatment are lacking and will be required to verify the cardiovascular efficacy/safety of chronic treatment.

Kelly and Jones [26] concluded that testosterone replacement in men diagnosed with hypogonadism, for whom mid-normal range levels are achieved, has a beneficial effect on several cardiovascular risk factors including cardiac ischemia, functional exercise capacity, and improved mortality. Yet studies in which patients were either undertreated or given high-dose testosterone have been associated with an increased risk of cardiovascular-related events. Clinical monitoring and titration of testosterone dose is therefore of paramount importance.

In a prospective study with testosterone undecanoate (TU), TU reduced fasting glucose, waist circumference, and improved surrogate markers of atherosclerosis in hypogonadal men with MS. Resumption and maintenance of T levels in the normal range of young adults determine a remarkable reduction in cardiovascular risk factors clustered in MS without significant hematological and prostate adverse events [27].

**Figure 4:**
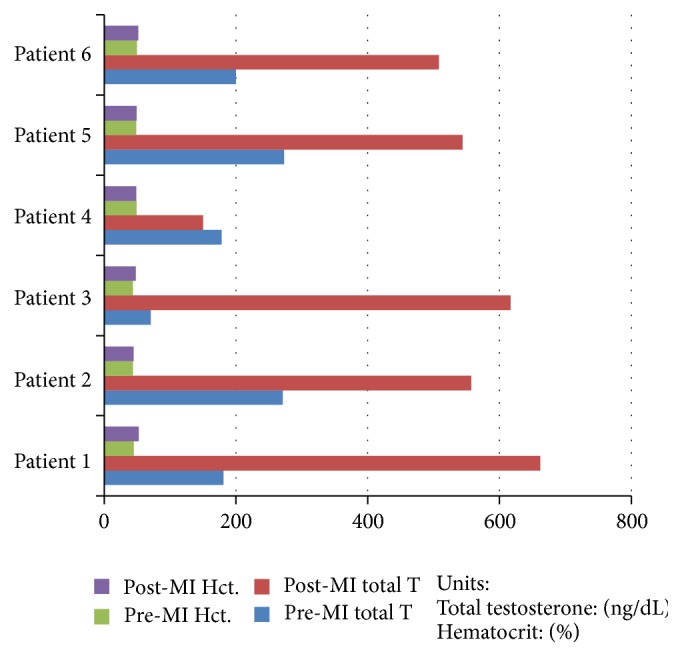
Changes of testosterone levels and hematocrit in patients with MI during treatment.

Our study reveals that MI does not appear to have a relationship with testosterone levels. Although the mean testosterone of patients with MI was lower by 62.5 ng/dL, we cannot infer that lower total mean testosterone levels led to the MI. The patients had established risk factors for MI. However, it may be fair to say that MI was not associated with higher levels of testosterone. Of the 6 cases with MI, the mean hematocrit was 48.97 and the mean initial hematocrit was 46.65 with a mean change of 2.32. There does not appear to be an association of MI to rise in hematocrit as the rise in patients without MI was similar or slightly higher, with mean change of 2.5 (Figure 4).

## 5. Conclusions

It appears from our study that practitioners should not only carefully screen and treat patients with hypogonadism but also carefully and closely monitor them. If patients are given T medications, compliance must be ensured. Only approximately 19,968 of our patients screened met criteria for treatment and were then followed on a protocol requiring regular, repeat clinic visits on a weekly or fortnightly basis for the prime modality of treatment with short acting injections. This strategy may have accounted for the positive outcomes that our study found. In addition, guidelines on treatment of hypogonadism (Endocrine Society, AACE, ISSAM, etc.) should be closely followed. It is important to ensure that patients reach therapeutic levels and monitor not only PSA and prostate but also the hematocrit. Our observation that the mean level of total testosterone was significantly higher in our study than the earlier study could be supportive of the association with low numbers of MI and stroke in testosterone-treated patients. Other comorbid risk factors such as diabetes, hyperlipidemia, and hypertension have also to be controlled, which appears to be the case with the close follow-ups, given our low rates of these comorbidities. Patients should be also given lifestyle change education. The flood of media advertisement may have pushed many patients into taking testosterone, but practitioners should not be reticent to deny treatment if patients do not qualify or have contraindications. It is important to spend time educating patients and telling them about the benefits and risks [28]. As no drug is completely safe for everyone, it is up to the practitioner to decide based on the understanding of risks/benefits by the patient and with informed consent.

Although the results of this study are promising, more research is recommended to further determine whether testosterone is cardioprotective or if it can cause MI or stroke in certain patient populations. A large randomized, controlled study powered in tens of thousands will be needed. To compare, the Women's Health Initiative had 161,800 patients, studied in a prospective manner [29].

These data suggest that treatment of low testosterone patients, particularly in the context of younger healthy men, does not lead to an increase of MI or stroke. This study population was considerably healthier even at baseline and may not be representative of the average hypogonadal patient. Furthermore, this study cannot claim that testosterone is protective against MI or stroke events given the lack of comparability of registries examined. In the Low T Center settings examined, use of testosterone was deemed safe, particularly for men under 65 years old without preexisting risk factors for heart attack or stroke. Low testosterone can be a public health issue, which, if left untreated, can lead to increased morbidity [30, 31].

## Figures and Tables

**Table 1:** Incidence rate of myocardial infarction (MI) from various registries (per 100,000 persons).

Study	Rate	Comments
United States National Hospital Discharge Survey, 2002	242	26-year study and noted case fatality rates decreased over time

New York State Registry (1996–2008)	71.6	13-year study and noted decrease mortality with time

Marshfield, Wisconsin, Epidemiology Study, 2002	292.4	6-year study of MI rates in stable population in WI

Fukushima Prefecture, Japan, 2013	37.9	Rates of MI were compared before & after the Tsunami

Kaiser Permanente, Northern California, 2008	208	The average of rates of MI from the 4 US registries approximates that of Kaiser at 203 per 100,000

Low T Centers, United States, 2014	30.0	Patients received testosterone injections

**Table 2:** Characteristics of Low Testosterone (T) Center patients in comparison to controls from other registry populations.

	Low T Centers	Kaiser Permanente	Northern Manhattan Registry	Comments
Male (%)	100	62	45	

<55 years (%)	87	Not reported	74	Kaiser reported age as 69 ± 14

>56 years (%)	13	Not reported	26	3% Low T > 65

White (%)	Not reported	67	22	Low T Centers did not collect ethnicity data

Black (%)	Not reported	7	13	

Hispanic (%)	Not reported	9	64	

Asian and others (%)	Not reported	17	1	

Hypertension (%)	15	76	Not reported	N Manhattan registry has high percentage of minorities which will imply higher rates of HTN

DM (%)	4	32	Not reported	N Manhattan registry has high percentage of minorities which will imply higher rates of DM

Hyperlipidemia (%)	12	80	Not reported	N Manhattan registry has high percentage of minorities which will imply higher rates of HLD

**Table 3:** Characteristics of study population from Low Testosterone (T) Centers.

	*N*	Percent
Patients on testosterone therapy	19,968	100%
Gender		
All male	19,968	100%
Age		
>55 years	3,833	19.2%
45–54 years	7,008	35.1%
35–44 years	6,829	34.2%
<34 years	2,296	11.5%
Prevalence of DM	798	4%
Prevalence of HTN	2,995	15%
Prevalence of HLD	2,396	12%
Mean age (years)	46.10	N/A
Drug		
Testosterone cypionate	18,742	93.8%
AndroGel	540	2.7%
Testim	326	1.6%
Fortesta	47	0.2%
Axiron	230	1%
Striant	9	0.04%
Others	74	0.66%
Hematocrit >52%	13,178	66%
Hematocrit ≤52%	8,785	44%

	Mean	Range

Time of exposure to testosterone (months)	24.7	0–60 month
Total testosterone level on treatment (ng/dL)	543 (SEM = 1.53)	50–1600
Calculated free testosterone on treatment (ng/mL)	13.08 (SEM = 0.48)	3.29–28.2
Estradiol level while on treatment (pg/dL)	31.1	6.4–453
PSA while on treatment (ng/mL)	1.22	0.02–109
